# Introducing the Automated Ligand Searcher

**DOI:** 10.1021/acs.jcim.3c01317

**Published:** 2023-11-20

**Authors:** Luise Jacobsen, Jonathan Hungerland, Vladimir Bačić, Luca Gerhards, Fabian Schuhmann, Ilia A. Solov’yov

**Affiliations:** † Department of Physics, Chemistry and Pharmacy, University of Southern Denmark, Campusvej 55, 5230 Odense M, Denmark; ‡ Institute of Physics, Carl von Ossietzky Universität, Carl-von-Ossietzky-Str. 9-11, 26129 Oldenburg, Germany; § Research Centre for Neurosensory Science, Carl von Ossietzky Universität Oldenburg, Carl-von-Ossietzky-Str. 9-11, 26129 Oldenburg, Germany; ∥ Center for Nanoscale Dynamics (CENAD), Carl von Ossietzky Universität Oldenburg, Ammerländer Heerstr. 114-118, 26129 Oldenburg, Germany; ¶ Niels Bohr International Academy, Niels Bohr Institute, University of Copenhagen, Blegdamsvej 17, 2100 Copenhagen, Denmark

## Abstract

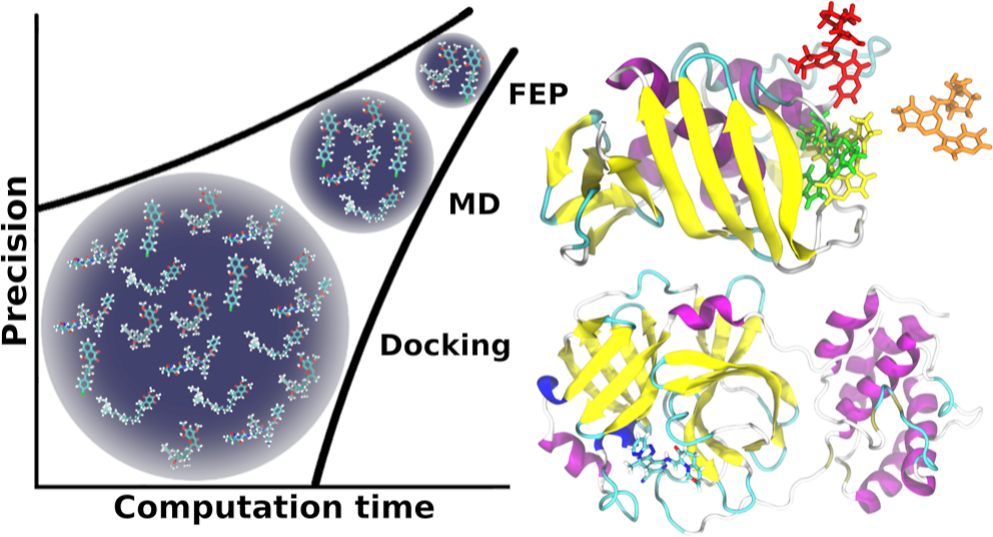

The Automated Ligand Searcher (ALISE) is designed as an automated computational drug discovery tool. To approximate the binding free energy of ligands to a receptor, ALISE includes a three-stage workflow, with each stage involving an increasingly sophisticated computational method: molecular docking, molecular dynamics, and free energy perturbation, respectively. To narrow the number of potential ligands, poorly performing ligands are gradually segregated out. The performance and usability of ALISE are benchmarked for a case study containing known active ligands and decoys for the HIV protease. The example illustrates that ALISE filters the decoys successfully and demonstrates that the automation, comprehensiveness, and user-friendliness of the software make it a valuable tool for improved and faster drug development workflows.

## Introduction

The emergence of deadly diseases and related epidemics has been a reoccurring phenomenon throughout recorded time. However, with the rapid increase in human population during the last couple of centuries and the resulting increased human–human and human–animal proximity, epidemics have become increasingly more frequent.^1^ Furthermore, due to international travel, diseases easily spread across countries, leading to global pandemics such as the HIV pandemic and the recent coronavirus disease 2019 (COVID-19) pandemic. Disease outbreaks have huge costs in human lives and the global economy. For example, COVID-19 is to date responsible for a death toll of more than 6.887 million people^2^ and is expected to cut the world’s GDP tremendously.^3,4^ Therefore, it is of critical importance that the current protocols used to combat diseases, for example, the development of testing methods, vaccines, and drugs, are improved and made faster.

The present paper focuses on suggesting improvements in drug discovery methods. The goal in drug discovery is to find a molecule (ligand) that binds to a target (receptor), typically a protein, and through the ligand–receptor interactions, blocks or modulates a particular biomolecular mechanism or pathway.^5,6^ In the case of infectious diseases, typically, mechanisms that are vital for the pathogen’s survival or reproduction are targeted.

The average cost of bringing one drug to market is estimated to be ∼1 billion USD.^7^ Clinical trials are the most cost-intensive parts of drug development (∼20%)^8,9^ due to toxicity tests. Furthermore, it has been estimated that only 0.01% of molecules synthesized or isolated during drug development are approved as pharmaceutical drugs. Hence, the large cost of developing a new drug is mainly due to the money and time spent on investigating unsuccessful candidates.^10^

To improve the identification of poor drug candidates early in the drug development process and, hence, optimize the efficiency of discovering a successful drug, it has become common practice to apply computational methods as part of the initial drug discovery.^11^ Molecular docking (docking) is a well-established method within computer-aided drug design^6,12,13^ that identifies the optimal binding pose of a ligand in a receptor and assigns to it a score based on its computed binding affinity. Existing computational docking programs screen thousands of ligands rapidly, but at the cost of a rather crude level of modeling. The most severe limitations are that only a limited number of chemical bonds in the ligands are flexible, and the receptor is often modeled as static or with only a few flexible residues.^6,14^ This highly constrained conformational search space may lead to a poor estimation of binding affinities. To improve computer-aided drug design, more refined methods need to be applied.^15^

In this paper, we present the three-stage Automated Ligand Searcher (ALISE) program, which, by applying increasingly refined computational methods in each stage, narrows down the number of potential drug candidates. The first stage applies the arguably primitive but fast docking method to discard all but the most promising ligands. It is important to note that since all ligands are initially screened in the docking stage, the overall tool is highly sensitive to the result of the docking algorithm. For the case study at hand, only 100 ligands were calculated in the molecular dynamics (MD) stage, which represents less than 0.3% of the complete ligand set. The remaining ligands proceed to the second stage, in which MD simulations are performed to obtain detailed insight into the interactions between the ligands and the receptor. Using binding free energy estimates obtained from MD simulations, the ligands with the strongest apparent binding free energies enter the third stage, where even more refined binding free energy estimates are obtained through advanced free energy perturbation (FEP) simulations.

To make the advantages of ALISE as accessible as possible, ALISE is implemented as a computational task in the versatile Scandinavian Online Kit for Nanoscale Modeling (https://viking-suite.com/VIKING) web platform^16^ which provides standardized step-by-step workflows for setting up and linking various computational modeling techniques, for example, MD simulations, FEP simulations, various quantum mechanical (QM) calculations, and so forth.^16−20^ Furthermore, VIKING connects to supercomputing clusters and allows one to run any task seamlessly on high-performance computing resources.

In the past, an earlier prototype version of ALISE has been successfully employed to study ligand–receptor binding in severe acute respiratory syndrome coronavirus 2 (SARS-CoV-2).^21^ Additionally, the ALISE framework can be used to structurally compare different receptor models and their ligand affinity. In the following, the theory and methods that form the basis of each stage in ALISE are outlined, and the ALISE workflow itself is presented. Furthermore, the capabilities of ALISE are demonstrated using the HIV protease for which a benchmarking set of active and decoy ligands are provided by the DUD-E database.^22^ The performance of ALISE to find a small number of active ligands in the vast amount of decoys is quantified.

## Methodology

**Figure 1:**
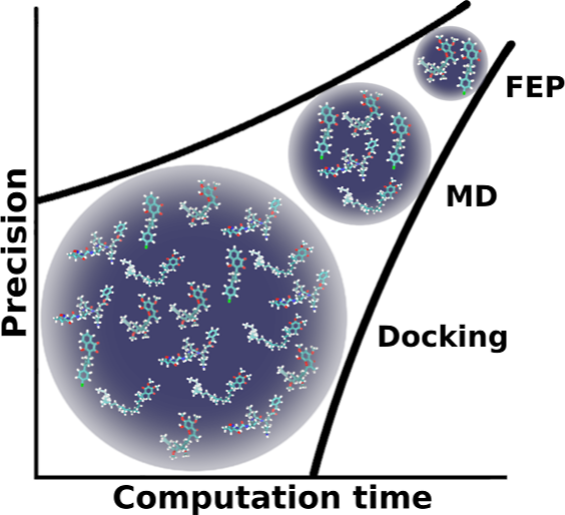
ALISE applies three consecutive stages, the molecular docking, MD, and FEP stages, to narrow and rank a list of potential drug candidates targeting a specified receptor. Each stage applies the increasingly sophisticated computation methods of molecular docking, MD simulations, and FEP simulations to estimate the binding free energy of the ligands to the receptor. To optimize the use of computational resources, only the best-ranked ligands from each stage proceed to the next, more sophisticated computational stage, and the list of ligands is gradually narrowed down to only the very best drug candidates.

From a collection of drug candidates, ALISE identifies the best candidates targeting a specific receptor through three consecutive stages: molecular docking, MD, and FEP. Figure 1 provides a schematic representation of how increasingly sophisticated modeling methods are employed in each stage to compute the binding free energies of putative ligand–receptor complexes, specifically molecular docking, MD, and FEP simulations, as illustrated by the three stages named in the figure. The estimated binding free energy of a ligand is referred to as its score, and separate molecular docking, MD, and FEP scores are obtained in their respective stages. At the end of each stage, only the ligands with the best scores are selected to proceed to the next stage. This gradual segregation of ligands in each stage ensures that computational resources are not wasted by applying more computationally expensive methods to study poor drug candidates. The basic theory and methods of each of the three stages in ALISE are presented in the following sections.

### Molecular Docking

Molecular docking is a modeling method utilized to determine the optimal binding pose, that is, the position, conformation, and orientation of a ligand at the binding site of a receptor, along with a docking score that measures the binding affinity of the ligand to the receptor in the determined binding pose.^14,23^ A typical docking software comprises two primary components: a search algorithm and a scoring function. The search algorithm moves the ligand through the conformational space defined by the degrees of freedom of the modeled system, while the scoring function ranks the docked configurations, which typically relies on an estimate of the free energy of the ligand binding to the receptor.^24^

The docking procedure in ALISE is carried out using the VinaMPI software,^25^ which is an extension of the AutoDock Vina (Vina) software with the addition of parallel MPI support for execution on supercomputers. Vina applies a hybrid scoring function *C*, derived from the empirical interatomic potentials,^14,23^ defined as
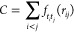
1where the summation is performed over all pairs of atoms whose interatomic distance, *r*_*ij*_, can vary.^23^ The function  describes the interaction between a pair of atoms of type *t*_1_ and *t*_2_, with the separation distance *r*_12_, as a weighted sum of steric, hydrophobic, and hydrogen bonding interaction terms, as described by Trott and Olson.^23^ The molecular docking procedure aims to find the global minimum of *C*, corresponding to the optimal binding pose of the ligand, which in Vina is accomplished by an iterated local search global optimizer^26^ which employs the Broyden–Fletcher–Goldfarb–Shanno method^27^ for local minimization.

To reduce the computational resource demand of searching the conformational space, only a restricted volume of the receptor, called the search space, is explored. The search space must be sufficiently large to cover the entire active site of the receptor without including unnecessary parts of the protein. Therefore, defining an appropriate search space becomes a mandatory part of the molecular docking procedure; a too-large search space wastes computational resources, while a too-small search space might exclude important parts of the conformational space.

### Molecular Dynamics

The second stage of ALISE determines the binding free energy of the investigated ligands based on equilibrium all-atom MD simulations performed using NAMD.^28,29^ In contrast to molecular docking, all parts of the considered systems are allowed to move during the MD stage. The atomic motions are obtained by integrating Newton’s second law for each atom with interatomic forces derived from molecular mechanics (MM) force fields obtained from experimental studies and QM modeling.^30^ Therefore, the MD stage provides a significantly more detailed and realistic description of the ligand–receptor binding process.

The ligand-binding free energy *G*^0^ is given by
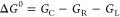
2where *G*_C_, *G*_L_, and *G*_R_ are the free energies of the ligand (L), receptor (R), and ligand–receptor complex (C), respectively. These energies are computed from the MD simulations as
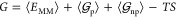
3where *E*_MM_ are the respective MM bonding and nonbonding energies,  and  are the polar and nonpolar contributions to the solvation free energy of the investigated structure, respectively, and *T* and *S* are the temperature and the entropy of the system. ⟨·⟩ indicates an average over the respective MD simulation trajectory.^31^

The first three terms in eq 3 are computed using the molecular mechanics-generalized Born and surface area continuum solvation (MM/GBSA) method.^31,32^ is proportional to the solvent-accessible surface area, *A*_SA_, of the investigated structure
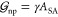
4where γ is a surface tension parameter.^33,34^ is computed using the generalized Born (GB) model, originally devised by Still et al.,^35^ but modified to take into account the ionization of the solvent^36−38^
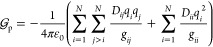
5where ε_0_ is the vacuum permittivity, summations are performed over all *N* atoms of the different subsystems (L, C, and R),^39^ and the function *g*_*ij*_ is defined as originally suggested by Still et al.^35^
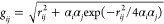
6where the effective Born radius of an atom, α_*i*_, indicates how deep an atom is buried inside a molecule or a protein;^40^ the deeper an atom is buried, the less accessible it is to the solvent, resulting in a larger value of α_*i*_.^39^ In ALISE, α_*i*_ is computed as proposed by Onufriev et al.^38−40^ and the calculation of *E*_MM_, , and  is controlled by the generalized Born implicit solvent (GBIS) functionality provided by NAMD,^38^ which combines the solvation free energy with the electrostatic energy output.

The term *D*_*ij*_ in eq 5 is defined as
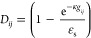
7where ε_s_ is the dielectric constant of the solvent, and  is the Debye screening length, with *k*_B_ being the Boltzmann constant, *N*_A_ the Avogadro number, *e* the elementary charge, and *I* the ion concentration within the GBIS.^38^ Note that the choice of an implicit solvent model limits accuracy in the MD stage. A hybrid model of explicit and implicit solvents, for example, as proposed by Geist et al.,^41^ would be beneficial.

In ALISE, the entropy is calculated using quasiharmonic analysis, which derives from a Gaussian approximation of the coordinates’ probability distributions and their interpretation as quantum harmonic oscillators, allowing the computation of the entropy from a principal component analysis.^42−46^ Within the outlined approximation, one estimates entropy as^42^
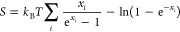
8
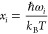
9where , with *h* being Planck’s constant, and the frequencies ω_*i*_ are obtained from the secular equation
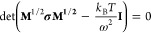
10where **M**^1/2^ is a diagonal matrix with the square roots of all atomic masses in the system, **σ** is the variance-covariance matrix of the Cartesian coordinates, calculated from the MD trajectory, and **I** is the identity matrix. The computed change in entropy upon ligand binding accounts for the total entropy of the ligand and the vibrational entropy of the receptor. The translational and rotational entropies of the receptor are not included since they can be expected to be constant during the binding event as long as the mass of the receptor is large compared to the mass of the ligand. Even if the latter is not the case, the often-desired inhibition of the active site by the ligand is independent of the rotational and translational mobility of the complex as long as the ligand is kept in its pocket. The rotational and translational contributions of the receptor are omitted by performing a coordinate alignment of the α-carbons of the receptor prior to the computation of the variance-covariance matrix.

### Free Energy Perturbation

In the final stage of ALISE, binding free energy estimates of the most promising ligands are computed by using FEP. FEP is also based on MD simulations, but in addition to simulating a system in only the bound and unbound states, as it is done in the MD stage, several intermediate states are simulated as well.^47,48^ Each simulated state is identified by a parameter λ_*i*_ ranging from 0 to 1, where *i* = 1, ..., *K* such that *K* is the total number of states. In a so-called forward transformation, the interactions between the ligand and its surroundings are gradually decoupled, described by increasing λ_*i*_ values, until the ligand is effectively annihilated at λ_*K*_ = 1. The free energy difference between each pair of simulated states, Δ*G*_*i*_, is computed by approximating the ensemble average following Zwanzig’s FEP identity^49^
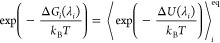
11by a direct average over simulation frames
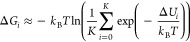
12where Δ*U*_*i*_ is the potential energy change associated with the change of the λ-parameter from λ_*i*_ to λ_*i*+1_ for a given set of atomic coordinates in the system. A total of *K* λ steps are performed. The total free energy change, Δ*G*, corresponding to the ligand annihilation is then obtained by summing over all Δ*G*_*i*_: Δ*G* = ∑_*i*=1_^*K*–1^Δ*G*_*i*_. By simulating the transformation of interest in small increments, FEP can, in principle, directly sample all the phase space changes related to the transformation between a bound and an unbound system, including all entropic contributions, and thereby measure the related free energy change. An analogous approach is performed to recouple interactions, defined as a backward transformation.

**Figure 2:**
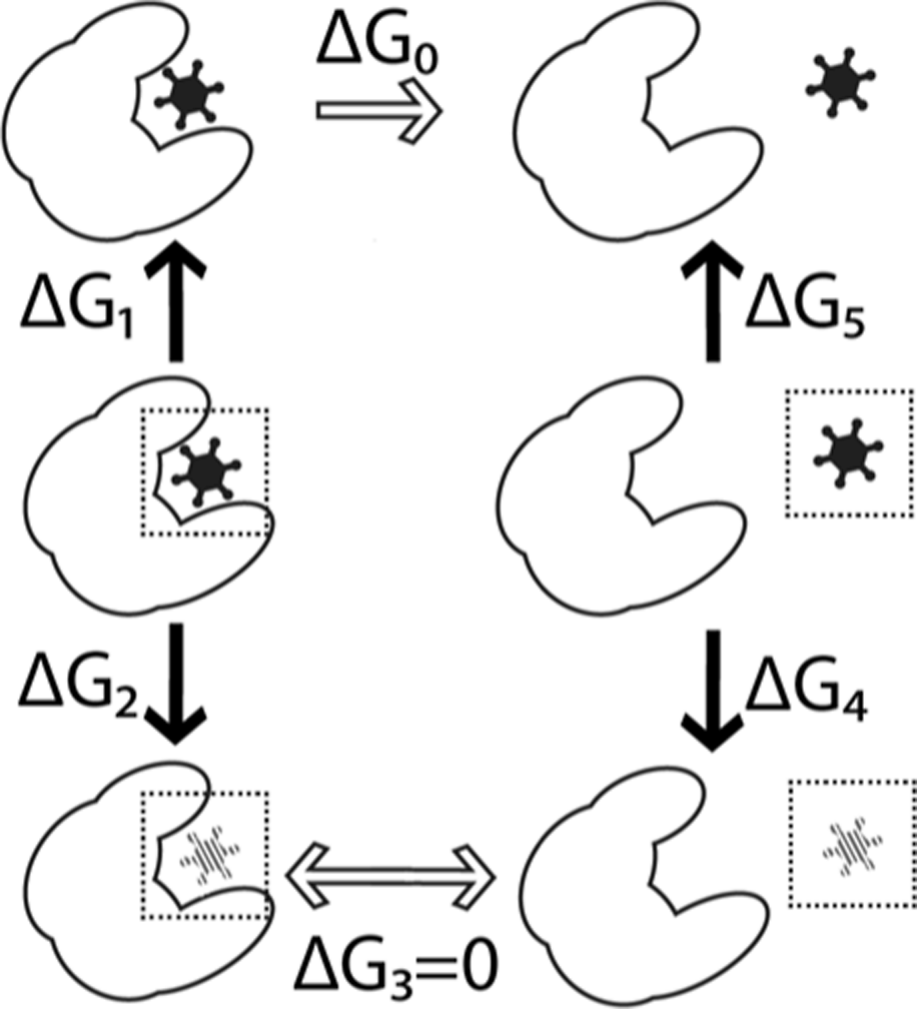
Illustration of the FEP cycle that is employed to calculate the binding free energy Δ*G*_0_ between the receptor (large shape) and the ligand (small shape). The existence of restraints on the ligand is represented by a dotted box around the ligand. When the ligand is colored solid black, it interacts with its environment, while the striped pattern indicates the absence of interactions with the environment. The FEP calculations are performed such that the forward perturbation is executed along the direction of the arrows.

In ALISE, four calculations, indicated by the solid arrows in Figure 2, are performed to obtain the transformation from a bound to an unbound state, from which the binding free energy can be estimated. For the unbound and bound states, two FEP simulations are performed: a forward and backward simulation. In order to keep the ligand in place and to avoid its collision with the receptor while interactions are gradually decoupled during the FEP calculation, several harmonic restraints are introduced. These include distance restraints, which anchor the ligand to selected non-hydrogen atoms of the receptor. Additionally, a restraint on the root-mean-square deviation of the ligand is imposed to keep the ligand from deforming. The restraints have a nonzero contribution to free energy and therefore must be considered in the calculation. Similarly to the forward and backward FEP simulations, the restraints are gradually coupled and decoupled in the bound and unbound configurations.

Since the ligand is not interacting with its surroundings in the lower part of the thermodynamical cycle in Figure 2, the cycle can be closed by setting Δ*G*_3_ = 0, as there is no difference in the free energy of the ligand embedded in water or the receptor. The binding free energy thus becomes
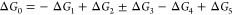
13

## Computational Realization of ALISE

**Figure 3:**
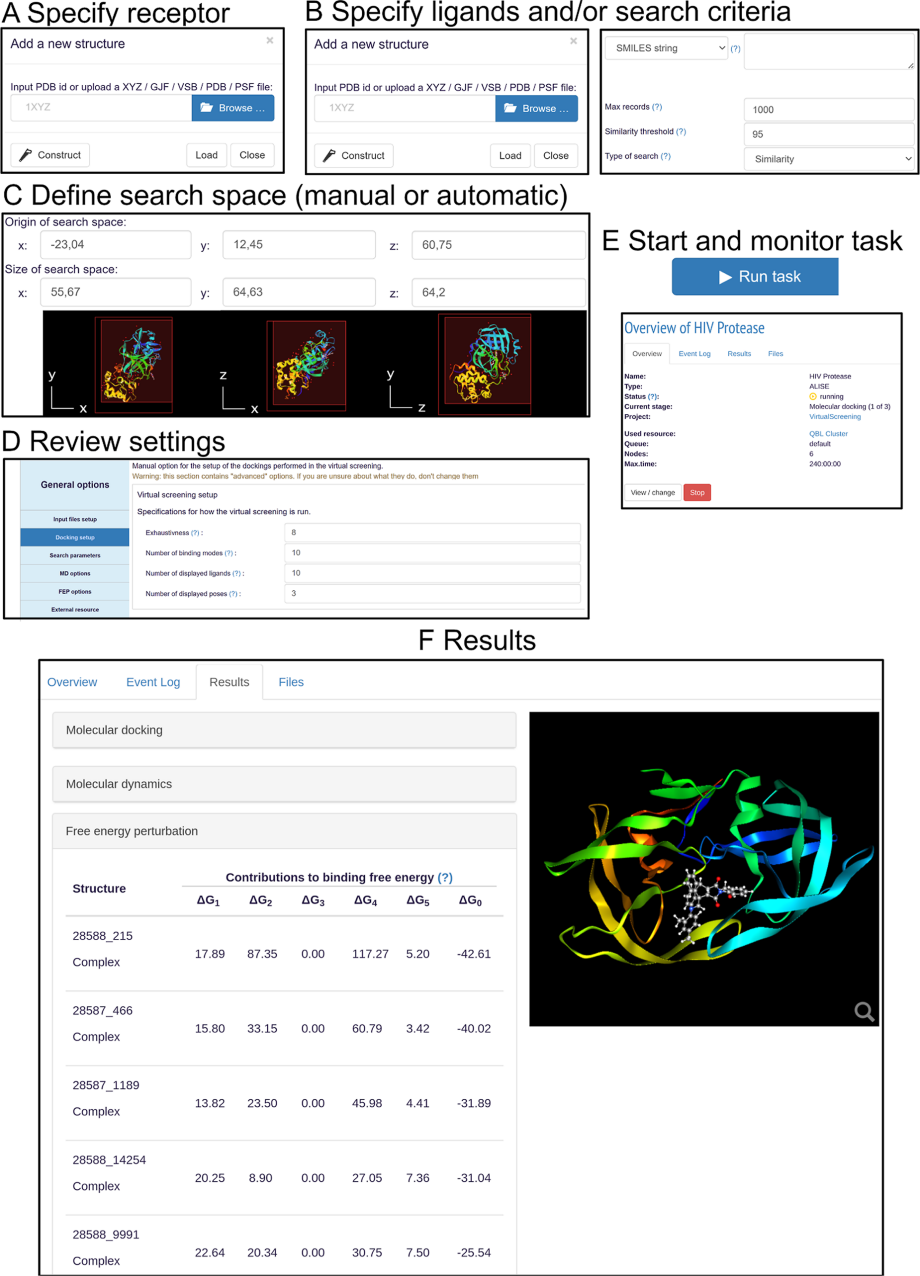
User interface of ALISE. (A,B) Molecular structures for the receptor and ligands can be uploaded or fetched from online databases.^52−54^ (C) The search space used in the docking stage can be defined manually or automatically based on the AutoLigand software.^55^ (D) On a summary page, all settings related to the docking and simulations performed during the virtual screening can be reviewed and modified. (E) After pressing “Run task”, the current status of the task can be monitored on the overview page. (F) As each stage completes, a result page will become available with a ranked list of the ligands based on their binding free energy estimates from that stage, along with graphical presentations of the modeled binding modes.

The web platform VIKING^16^ offers access to the ALISE software, which provides a user-friendly workflow interface similar to other computational tasks in VIKING.^17−20^ In order to set up a virtual screening experiment, the user is prompted to provide specific information. Steps A-C, as shown in Figure 3, require the following information:

(A) Target receptor: The user can upload a molecular structure file of the receptor or provide a Protein Data Bank (PDB) ID to automatically search for the receptor in the database.

(B) Ligands: Potential ligands can be uploaded as molecular structure files, or the system can retrieve them automatically from the PubChem database^52,53^ through a chemical similarity search. The similarity is assessed through the Tanimoto score (see Supporting Information: Chemical Similarity Search on PubChem). Additional screening for possible toxicity is possible by utilizing toolkits^21,50^ that calculate the quantitative estimate of drug likeness^51^ and uploading their output as a list of mol2 files.

(C) Search space: The user can either manually set the dimensions and location of the search space or use a graphical user interface in VIKING to drag a search space box. Alternatively, ALISE can automatically determine the search space using the AutoLigand software^55^ (see Supporting Information: AutoLigand).

Once these initial steps are completed, the user reaches a summary page (step D in Figure 3), where all of the settings can be reviewed and adjusted, including those specific to each of the three stages used in a virtual screening experiment. By default, all settings are set to sensible values. However, expert users have the flexibility to adjust virtually every aspect of ALISE. Notably, the docking exhaustiveness and simulation parameters, such as simulation length, time step, and output frequencies of the MD and FEP stages, can be adjusted. Furthermore, the number of intermediate alchemical states to simulate during the FEP stage can be changed to control the statistical reliability of the obtained free energy estimates. A detailed description of all available settings in the user interface is available in Supporting Information (virtual screening setup options).

**Figure 4:**
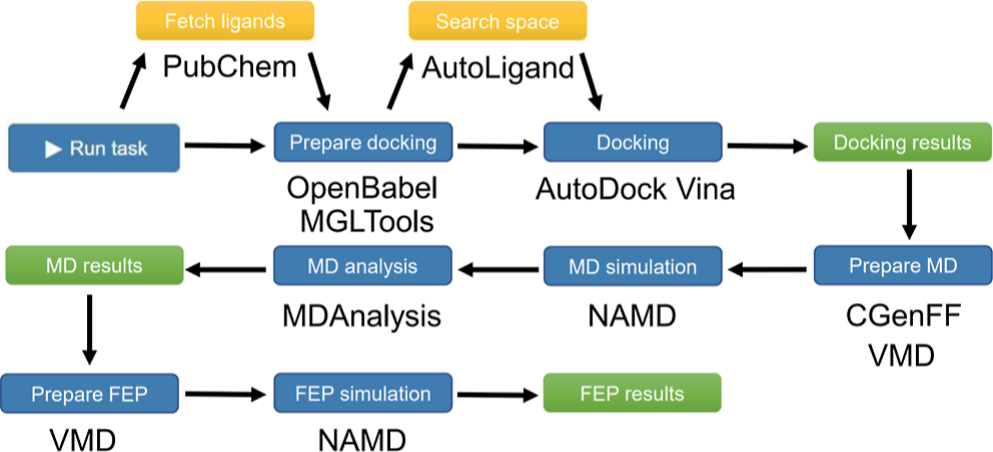
Automated workflow executed by ALISE. Optional and mandatory steps are represented by yellow and blue boxes, respectively, while green boxes indicate results obtained after each stage. Results are depicted on the result pages; see step F in Figure 3. Names^56−60^ indicate the software or web resource utilized by ALISE in the particular step of the workflow.

The search for suitable drug molecules is initiated from the task summary page on the VIKING platform. VIKING then launches the virtual screening, and the transitions between the steps are automatically taken care of. The progress of a running experiment can be monitored on the overview page (see step E in Figure 3) from which intermediate results and files are also available. A screen capture demonstrating the process of setting up a task in ALISE can be found via the Data and Software Availability statement. Figure 4 outlines the automated workflow handled by ALISE when the task is executed. First, if requested, ligands are fetched from the PubChem database^52,53^ after which the receptor and ligand files are prepared for docking using AutoDockTools^61^ and OpenBabel.^62^ Next, if needed, a suitable search space is determined using AutoLigand.^55^ Docking is performed by VinaMPI^25^ and a user-defined number of best-scoring ligands and poses are presented on the result page, see step F in Figure 3.

For each of the best-performing ligands from the docking stage, a set of MM force field parameters is automatically generated by the Charmm General Force Field (CGenFF) program,^63−65^ and MD simulations employing the implicit solvent approximation are prepared and initiated based on the determined binding poses. Both in the MD and FEP stages, the simulation files are prepared using the psfgen plugin^66^ in VMD,^67^ and the simulations are performed using NAMD.^28,29^ For *N* ligands, a total of 2*N* + 1 simulations are prepared and executed in the MD stage: one simulation with the receptor, one with each ligand, and finally one simulation with each receptor–ligand complex. When the MD simulations are done, the free energy contributions are computed for each simulated system following eq 3, and the differences in the free energy contributions are provided on the result page (see step F in Figure 3) together with the total binding free energy estimates computed following eq 2.

In the last stage, FEP simulations are prepared and executed from the last configurations of the best-performing ligands obtained from the MD stage, employing the explicit solvent model. Here, the solvate^68^ and autoionize^69^ plugins of VMD^67^ are used, respectively, to position structures in water boxes with a user-specified distance to the water box boundaries and to add Na^+^ and Cl^–^ ions to the system, matching the concentrations used during the MD stage. The free energy difference obtained from decoupling the ligand from the complex, see Figure 2 and eq 13, is finally determined and summarized on the result page (see step F in Figure 3). In all simulations, proteins, solvents, and ions are modeled by the Chemistry at Harvard Macromolecular Mechanics (CHARMM) additive all-atom force fields with CMAP corrections.^70−72^ The inclusion of other force fields, for example, AMBER,^73^ is planned for the future development of ALISE.

## Proof of Concept and Discussion

One protein that has received significant attention in recent years and remains a major challenge in human medicine is the human immunodeficiency virus type 1 (HIV-1) protease.^74−76^ Millions of people are infected with HIV^77^ and the occurrence of drug-resistant virus variants^78,79^ requires researchers to respond. Identifying biologically active ligands that can bind to the protease and hinder the replication cycle of HIV is an essential and significant task. Docking algorithms can assist in this process.

Quantifying the precision and efficancy of a docking algorithm is, however, a complex procedure that has been extensively discussed^80−82^ and the demand to validate different kinds of approaches is growing.^83^ In recent years, the DUD-E (Directory of Useful Decoys-Enhanced) database^22^ has become a reliable benchmark library for assessing the performance of docking algorithms. The DUD-E database contains a combination of active ligands, which are biologically active molecules or compounds that interact with a target protein and exhibit desired pharmacological effects, and decoy ligands, which are nonbiologically active molecules included as negative controls during molecular docking experiments. Using data sets from the DUD-E database, it is possible to evaluate whether a docking algorithm effectively identifies active ligands without falsely including decoy ligands.

### Performance

We have used the DUD-E data set for HIV-1 protease to benchmark the performance of ALISE to determine if the program is capable of identifying more active ligands for the protease compared to a random selection. Additionally, we compare and discuss the three different steps involved: docking, MD, and FEP.

The data set used in this study comprises 1,395 active ligands and 35,750 decoys. ALISE aims to sort these ligands, favoring the active ones. In each step, ALISE generates an ordered list of ligands based on their scores, and the top-scoring ligands progress to the next stage. Ligands that fail to dock or be simulated are ranked as the worst. Possible reasons for failure include unsuccessful docking when VinaMPI cannot fit the ligand in the defined binding site, CGenFF failing to produce parameters, or an unstable MD simulation. The latter occurred only once and was caused by unphysical parameters, leading to sudden and large accelerations. The specific parameters involved a sulfur atom, to which CGenFF assigned large penalty scores of about 100. A complete report is included in the replication data; see the Data and Software Availability statement. At each stage, the performance of ALISE is evaluated and compared to a random selection of the ligands from the data set.

**Figure 5:**
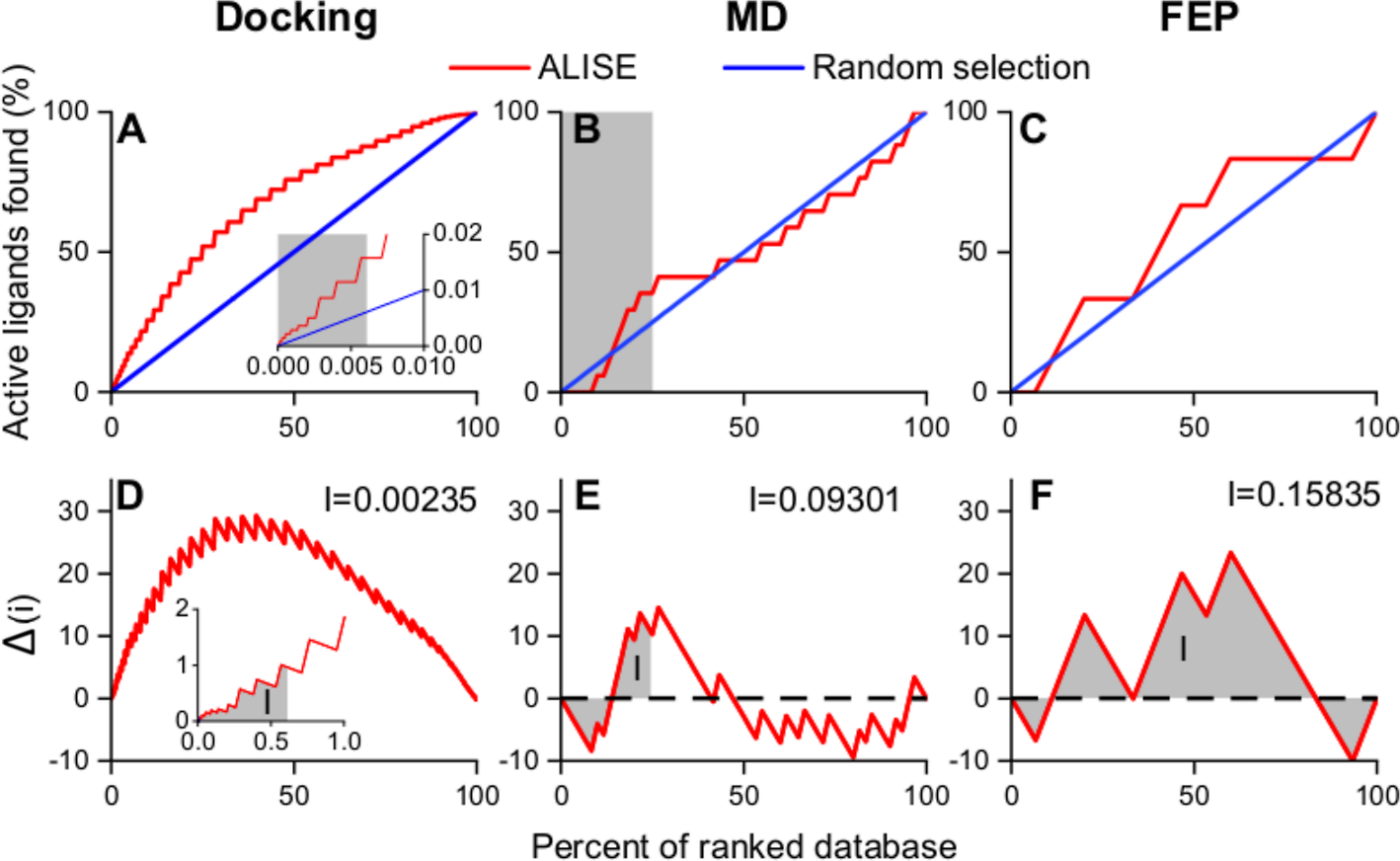
Comparison of ALISE’s performance in finding active ligands compared to a random selection at the three computational stages. The abcissa represents the progress in percent through all ligands (active and decoys), while the ordinate shows the percentage of active ligands found within this fraction (first row). In the second row, the ordinate shows the difference between the curves. The individual stage has a better performance if the red curve (ranked by ALISE) is above the blue curve (ranked by random selection). (A) Results of the docking stage show ALISE’s ordering (red line) surpassing the random selection (blue line) consistently for all ligands, including the successful docking of 1395 active ligands (last point). The gray area represents 100 ligands proceeding to the MD stage. (B) Results of the MD stage show that some active ligands are found more efficiently with ALISE compared with random selection. The gray area represents the 15 ligands selected for the FEP stage. (C) Results of the FEP stage further improve the results of the MD stage. The FEP stage ranks the active ligands more favorably than the random selection procedure. (D–F) For each respective stage, the difference between ALISE’s performance and the random selection approach is shown. The integrals *I* (grayed areas) are a measure of ALISE’s supremacy over random selection. A positive value indicates ALISE’s advantage in performance. For the docking stage, the value is 0.00235; for the MD stage, a value of 0.0931 was found; and for the FEP stage, 0.15835 was calculated.

Figure 5A–C illustrates the comparison of the virtual screening results obtained using ALISE versus employing a random selection of ligands at each stage of the ligand docking process. Each value *x* on the abcissa represents the top *x* percent of the ranked database, and the ordinate quantifies the percentage of known active ligands that are found within the top *x* percent of the ranked database, resulting in so-called enrichment curves. The difference between the curves obtained through ALISE (red) and random selection (blue) describes the effectiveness of each computational step and is visualized in Figure 5D–F.

ALISE outperforms the random selection if the integrals indicated in Figure 5D–F are positive. The docking stage shows an obvious supremacy in sorting. On the other hand, the benefits of the MD and FEP seem to be subtler. Integration of the curves in 5D–F, as indicated by the gray area, yields values of 0.00235 for the docking stage, 0.09301 for the MD stage, and finally 0.15835 for the FEP stage. As all values are positive, the ALISE workflow provides a quantifiable benefit in each stage compared to a random choice of ligands. In the following, each stage is discussed in more detail:

In the docking stage, a total of 16,329 ligands were successfully docked, including all 1395 active ligands (see Figure 5A). The chance to randomly select an active ligand in this stage is 3.90%. From the docking results, the top 100 ligands based on their docking scores proceeded to the MD stage, represented by the gray area in Figure 5A.

During the MD stage, 39 simulations failed, including three active ligands. In most cases, CGenFF could not produce parameters for the ligand. A more detailed status report is given in a supporting .csv file; see the Data and Software Availability statement. Figure 5B shows that at the MD stage, some active ligands were favored, but the overall performance of ALISE was similar to the random selection of ligands with lower scores. Nevertheless, the MD stage has a positive effect, as seen in Figure 5B,E. Furthermore, the chance to randomly choose an active ligand is now 27.86%, as opposed to 3.90% in the docking stage, requiring a more elaborate method to keep outperforming random choice.

Considering the resource demands, only the top 15 ligands from the MD simulations were selected for further analysis in the FEP stage, as indicated by the gray area in Figure 5B. The results in Figure 5C demonstrate that at the FEP stage, the findings of the MD step are improved, and active ligands are discovered more swiftly compared to a random selection. At the FEP stage, the chance to randomly choose an active ligand has already increased to 40.00% in the random selection.

The final stage of the overall ALISE workflow yields an ordered list and provides a visualization of the respective receptor–ligand complexes within the VIKING visualization framework. In summary, while only 3.90% of the ligands tested were active, 40.00% of the ligands tested in the final stage are active ligands. Additionally, in the final stage, the majority of decoy ligands were ranked lower than the active ones. All calculations were performed on a single node with 48 CPUs and a 2.9 GHz frequency. Choosing 100 ligands to be considered in the MD stage and 15 ligands in the FEP stage ensured that all jobs completed within 2 weeks on the available resources. The docking stage took, on average, 0.5 s per docked position. For the MD stage and FEP stage, performances of 2.08 and 4.01 h/ns were observed, respectively.

### Binding Results

The workflow implemented in ALISE permitted the reduction of the number of putative ligands to bind to the HIV protease from 35,750 to just 15. The following section provides a more detailed inspection of the top three highest-ranked ligands, as delivered by the FEP stage of the program.

**Figure 6:**
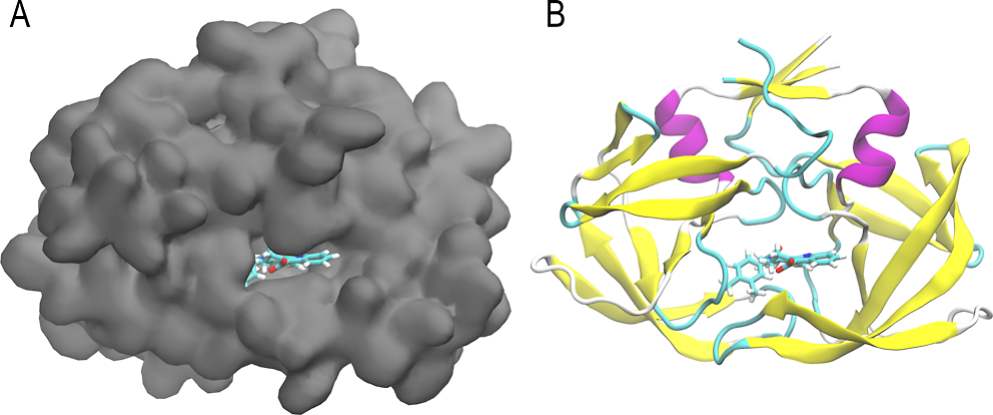
Panels A and B show the ligand with the best binding free energy estimate, as obtained through the ALISE framework. A shows the bound ligand on the surface of the protein structure, while B shows the secondary structure of the protein.

**Figure 7:**
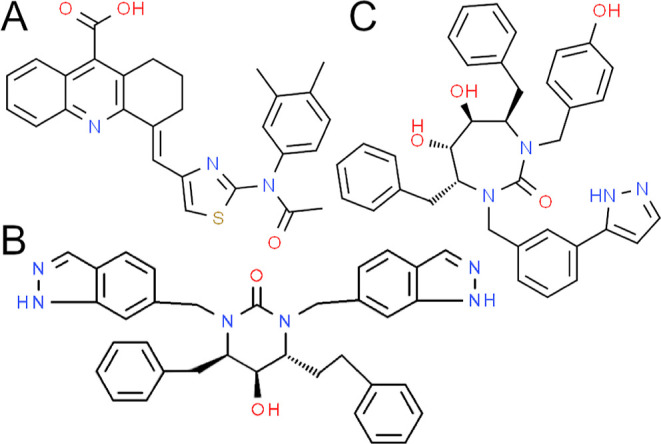
Panels A–C show the structures of the three highest-ranked ligands found by ALISE to inhibit the HIV protease. Structures are obtained from the ChemSpider Web site.^87−89^

The overall highest-ranking ligand is a decoy, according to the DUD-E database. It was ranked with an estimated binding free energy of −42.60 kcal/mol and is shown in its bound configuration in Figure 6. To the best of our knowledge, this decoy ligand has not been experimentally studied or mentioned to have certain effects. It might therefore be that the ligand is not stable enough in nature or was not studied sufficiently to make any definite conclusions. It is, of course, also possible that a new putative drug to inhibit HIV protease has been identified. The molecular structure of the ligand is shown in Figure 7A.

The second highest-ranked ligand (PubChem CID 471313) has a binding free energy estimate similar to that of the first ligand, with a value of −40.02 kcal/mol. The ligand can be categorized as a tetrahydropyrimidinone. DeLucca et al.^84^ demonstrated that molecules of that kind bind well in the HIV protease binding pocket by displacing structural water molecules. The structure of the ligand is shown in Figure 7B.

For the third highest-ranked ligand, ALISE obtained a binding free energy estimate of −31.89 kcal/mol. It is an active compound, as determined by the DUD-E database. This particular ligand (PubChem CID 469254) is a derivative of the cyclic urea inhibitor DMP450. Previous research has demonstrated that this class of compounds can effectively inhibit the activity of the HIV protease enzyme through specific bonding interactions.^85^ Notably, the ligand incorporates hydrogen-bonding equivalents of a water molecule that is typically bound to the enzyme, resulting in the formation of a conformationally rigid, seven-membered ring.^84^ These molecular characteristics contribute to the optimal interaction between DMP450 and all of the binding pockets of the HIV protease enzyme. The ligand is an experimentally synthesized asymmetric derivative of DMP450. It was named the 12F structure by Han et al.,^86^ and its structure is illustrated in Figure 7C.

The binding score corresponding to the estimated free energies of the first 200 ligands from the docking stage and all ligands in the subsequent stages are listed in the Supporting Information (ALISE’s ranked results for the HIV protease case study).

## Conclusions

ALISE is an advanced virtual drug screening tool integrated into the versatile https://viking-suite.com/VIKING web platform.^16^ The primary objective of ALISE is to refine and condense a preliminary list of potential drug candidates targeting a specific receptor. This is achieved through a sequential process consisting of three distinct stages, wherein the ligands are ranked based on their binding free energy estimates. The binding free energy estimates are computed by using three different computational techniques: docking, MD simulations, and FEP simulations.

At each stage, only the ligands exhibiting the most favorable binding free energy values proceed to the subsequent stage. This stepwise segregation of ligands ensures optimal utilization of computational resources by avoiding the application of computationally demanding methods to unpromising drug candidates. The results obtained after each stage, including the binding free energy estimates and a graphical representation of the outcomes, are made accessible on a dedicated result page within the VIKING online platform, as indicated in Figure 3.

To demonstrate the performance of ALISE, a benchmark example was conducted that focused on the HIV protease. The benchmark involved a data set with less than 4% active ligands, and ALISE successfully narrowed the search to 40% of active ligands in the final stage with 15 ligands. Furthermore, two out of the three highest-ranked drugs were successfully tested to inhibit HIV in earlier investigations.^84,86^

Overall, ALISE’s automated and systematic approach to virtual drug screening combines 10 individual programs and automates their application in three stages. The automated approach, the web resource implementation, and the performance show that ALISE has potential as an efficient and effective tool in the early stages of drug discovery and optimization. ALISE is a user-friendly tool which allows computational scientists to use a virtual screening workflow. At the same time, ALISE speeds up the workflow for experts in the field.

## Data Availability

The data needed to reproduce this study, a screen capture showing the setup of a task in ALISE, and a status report from the study are publicly available at: https://doi.org/10.57782/LGTW2K. The data structure is described in a README file. Software, web servers, and computational tools applied in ALISE and presented in this paper are owned by their respective developers and copyright holders but can be licensed freely for academic use. Except for the CGenFF program, VIKING redistributes the necessary software and tools to users’ computational resources. To ensure that users of ALISE are noncommercial and possess a CGenFF license, each user must apply for access to ALISE.

## Supporting Information Available

Chemical similarity search on PubChem; AutoLigand; virtual screening setup options; and ALISE’s ranked results for the HIV protease case study (PDF)

## Abbreviations Used

ALISE: Automated Ligand Searcher
CGenFF: Charmm General Force Field
CHARMM: Chemistry at Harvard Macromolecular Mechanics
COVID-19: coronavirus disease 2019
FEP: free energy perturbation
GB: generalized Born
GBIS: generalized Born implicit solvent
MD: molecular dynamics
MM: molecular mechanics
MM/GBSA: molecular mechanics generalized Born and surface area continuum solvation
QM: quantum mechanical
Vina: AutoDock Vina
