# Supplementary material for: Introducing the Automated Ligand Searcher
Source: J Chem Inf Model. 2023 Nov 20;63(23):7518–28. doi: 10.1021/acs.jcim.3c01317 (PMC10716895; doi:10.1021/acs.jcim.3c01317)
Supplement: Supplementary file 1 — ci3c01317_si_001.pdf [file ci3c01317_si_001.pdf]

# Supporting Information
# Introducing the Automated Ligand Searcher (ALISE)

Luise Jacobsen,[†,||] Jonathan Hungerland,[‡,||] Vladimir Bačić,[‡] Luca Gerhards,[‡]
Fabian Schuhmann,[*,‡] and Ilia A. Solov'yov[*,‡,¶,§]

†*Department of Physics, Chemistry and Pharmacy, University of Southern Denmark, Campusvej 55, 5230 Odense M, Denmark*

‡*Institute of Physics, Carl von Ossietzky Universität, Carl-von-Ossietzky-Str. 9-11, 26129 Oldenburg, Germany*

¶*Research Centre for Neurosensory Science, Carl von Ossietzky Universität Oldenburg, Carl-von-Ossietzky-Str. 9-11, 26129 Oldenburg, Germany*

§*Center for Nanoscale Dynamics (CENAD), Carl von Ossietzky Universität Oldenburg, Ammerländer Heerstr. 114-118, 26129 Oldenburg, Germany*

||*Contributed equally to this work*

* E-mail: fabian.schuhmann@uni-oldenburg.de; ilia.solovyov@uni-oldenburg.de

# Contents

**Chemical Similarity Search on PubChem** **S3**

**AutoLigand** **S3**

**Virtual Screening Setup Options** **S6**

**ALISE's ranked results for the HIV protease case study** **S10**

**References** **S15**

## Chemical Similarity Search on PubChem

Automated Ligand Searcher (ALISE) can search for potential ligands from the PubChem database[1,2] through the PubChem Power User Gateway - Representational State Transfer (PUG-REST) interface.[3,4] This search is based on chemical similarity, which can be described as follows. A molecule can be characterized by a binary string (molecular fingerprint) whose bits identify whether it contains a specific molecular fragment (1) or not (0).[5] The percentage of similarity between two ligands with molecular fingerprints $A$ and $B$ is determined by a Tanimoto score, $K$, which is the fraction of the intersection and union of fragments in the molecules, identical to 1s in $A$ and $B$:[6]

$$K = \frac{|A \cap B|}{|A \cup B|}. \tag{S1}$$

To fetch the ligands similar to the given input ligand, the user must supply its molecular formula or simplified molecular input line entry system (SMILES) string,[7] as well as the similarity threshold and the number of desired ligands.

## AutoLigand

Based on the ligand with the most rotatable bonds and the receptor, ALISE can automatically determine a suitable search space using AutoLigand.[8] AutoLigand identifies the continuous volume in the receptor with the highest interaction energy. A grid with 1 Å spacing that covers the entire receptor is created using AutoGrid4[9,10] and stores in its grid points the potential between the receptor and any atom type in the ligand[8] in that grid point.

AutoLigand performs three steps: flood fill, local migration, and ray-casting neighborhood search.[8] The flood fill is initiated from one grid point and new points are sequentially added to the neighbor grid point with the highest potential until a continuous collection of grid points (an envelope) of a predefined size is formed. Flood fills are initiated from every

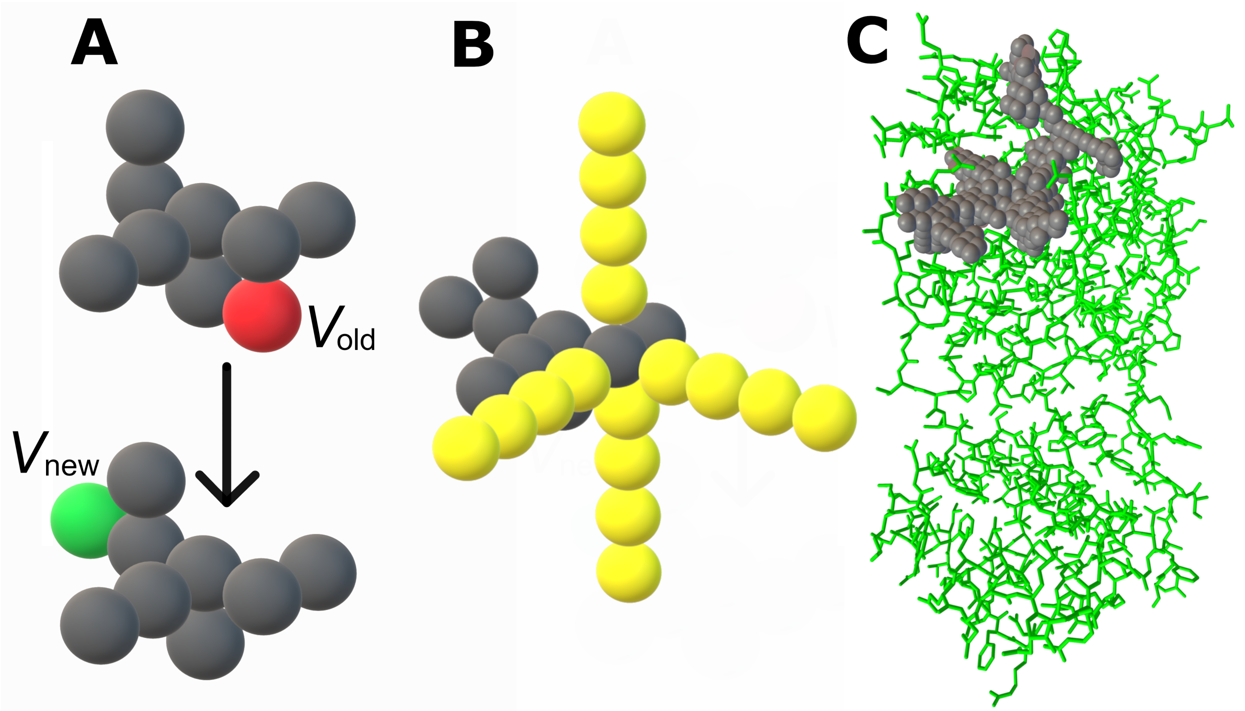


Figure S1: The AutoLigand method. Possible ligand binding sites on the receptor are represented by collections of grid points (envelopes). **A**) In a migration step, the interaction energy of an envelope is improved by substituting low-potential points (red) with higher potential neighbor points (green). **B**) In the following step the neighborhood is investigated by casting rays of points (yellow) from each point in the envelope. If the search improves the potential of the envelope, a ray substitutes existing points in the envelope. Migration and ray casting are repeated until an envelope of converged potential is found as illustrated in **C**.

fourth grid point unless the grid point is part of an existing flood fill.[8]

In local migration, the ten envelopes with the highest accumulated potentials are optimized by substituting the point in the envelope with the lowest potential, $V_{\mathrm{old}}$, with that of the neighbor grid point of highest potential, $V_{\mathrm{new}}$, see Fig. S1A. Substitution is allowed only if $|V_{\mathrm{new}}| > |V_{\mathrm{old}}|$ and the envelope remains continuous. Local migration stops when no substitution will improve the potential of the envelope.

The ray-casting neighborhood search investigates a larger neighborhood for better potential. Rays of up to ten grid points are cast in six directions from all points in the envelope, see Fig. S1B. If the points in a ray have higher potential than the same number of lowest potential points in the envelope, the low-potential points are substituted by the points in

the ray. The migration and ray-casting steps are repeated until an envelope of converged potential is found as illustrated in Fig. S1C.

# Virtual Screening Setup Options

In ALISE, a virtual screening experiment can be performed by only supplying a receptor and ligands of interest. However, to leave expert users as much flexibility as possible, virtually every aspect of ALISE can be adjusted from the summary page. The summary page is divided into six sub-menus:

**General options.** A summary of the settings requested in the preliminary steps of ALISE, presented in Fig. 3 and the section Computational Realization of ALISE in the main paper. Additionally, it is here possible to switch between different computational resources.

**Input files setup.** Figure S2 shows the input files setup options. Here it can be chosen which types of molecular bonds should be flexible in the ligands, and if any amino acid side chains should be flexible during docking. Furthermore, it is possible to choose whether the receptor should be cleaned for water molecules and irregular residues, i.e., non-amino acids.

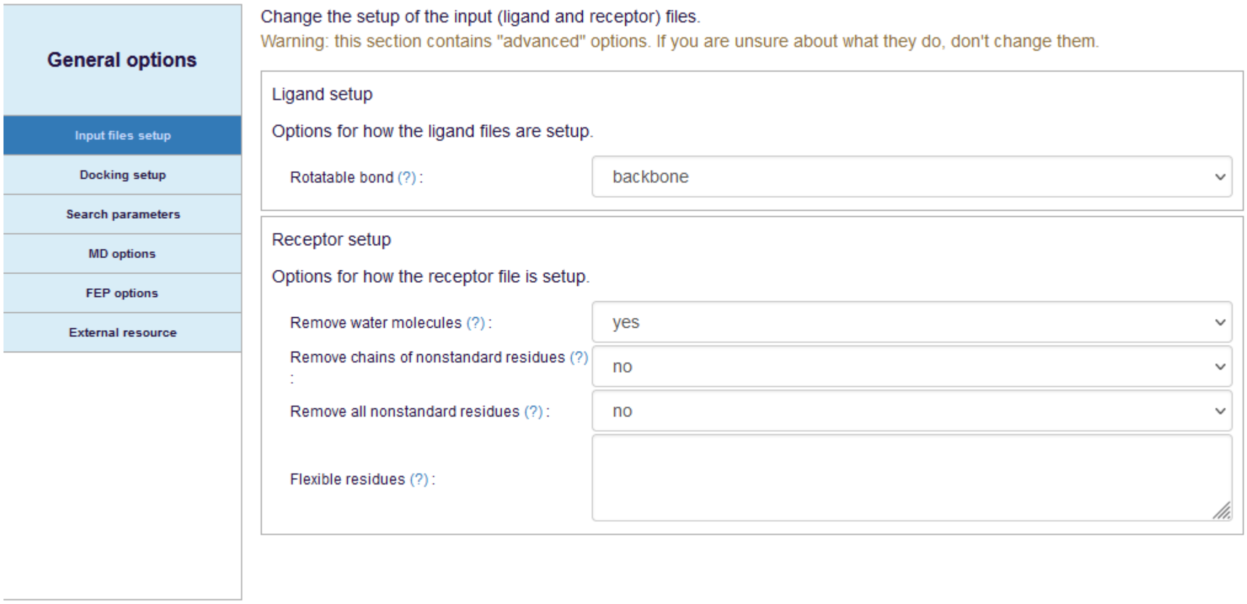


Figure S2: Sub-menu allowing to modify how the receptor and ligand files are set up.

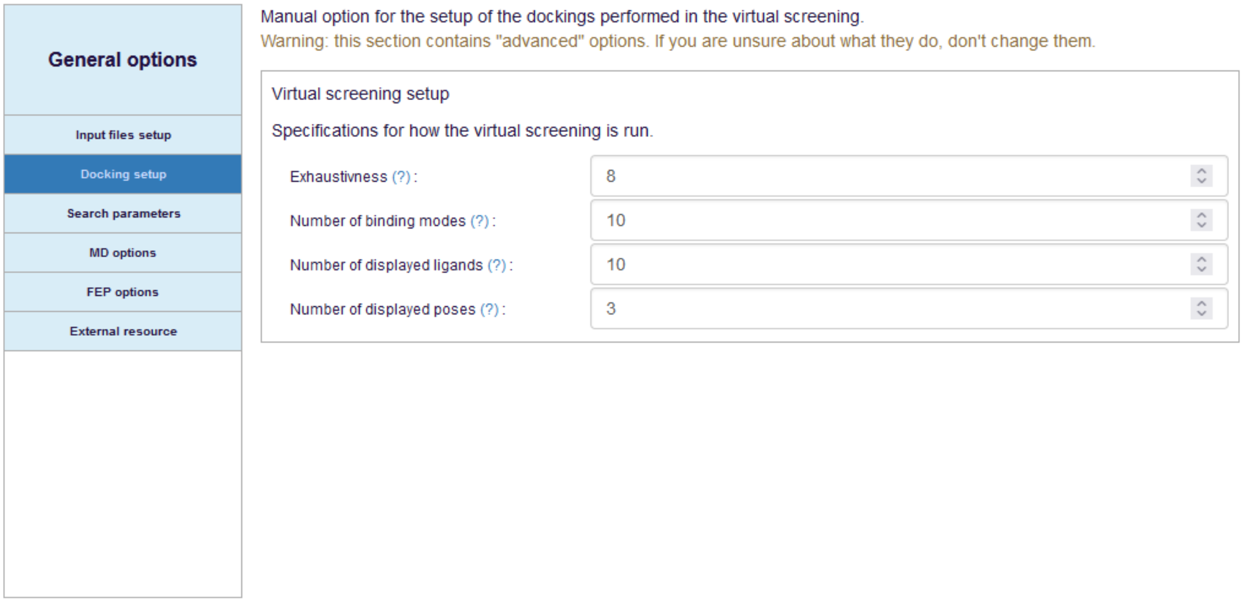


Figure S3: Sub-menu allowing to modify how the docking is performed and how many docked ligands to present on the result page.

**Docking setup.** Figure S3 shows the docking setup options which include specifications for how exhaustive the docking should be and how many ligand poses should be generated per ligand. It is also here that the user specifies how many ligands and ligand poses to present on the result page.

**Search parameters.** Revisits the search criteria used to search for ligands on the PubChem database,[1,2] see step 2 of Fig. 3 in the main paper.

**MD options.** Figure S4 shows the MD setup options. The parameters used by the generalized Born implicit solvent functionality to compute solvation free energies can be changed. Furthermore, standard MD settings: simulation time, time step, and system temperature, as well as the criteria for calculating interaction energies, e.g., the cutoff and switching distance, can be updated here. Finally, output frequencies for trajectory and energy files, and the number of MD simulations to perform in the virtual screening experiment can be modified.

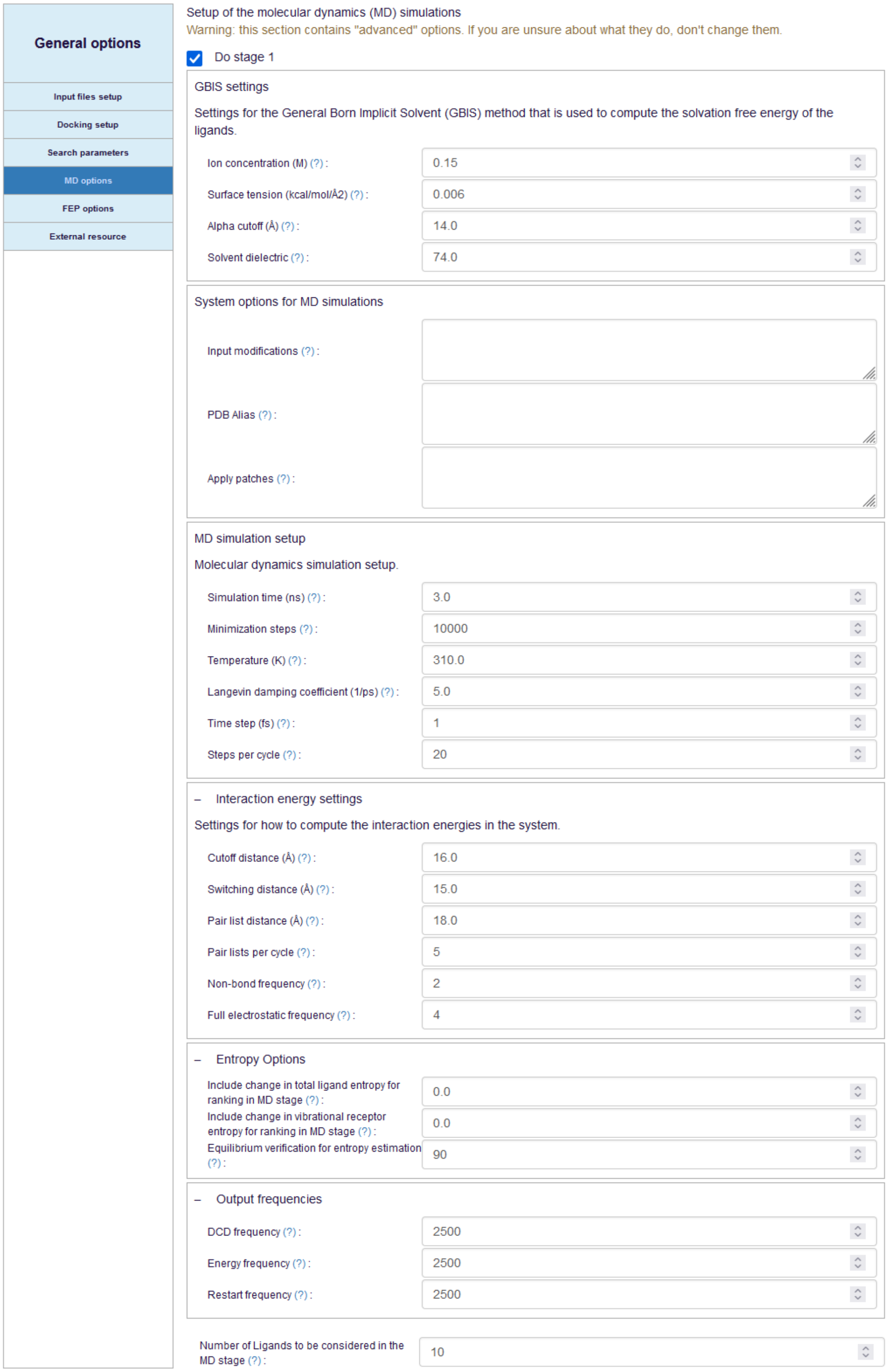


Figure S4: Sub-menu allowing to modify how the molecular dynamics (MD) simulations are performed. The options affect how solvation free energies and interaction energies are computed. Furthermore, the simulation length and resolution can be modified here.

**FEP options.** Figure S5 shows the free energy perturbation (FEP) setup options. The options include how many equilibration and simulation steps to perform in each step of the FEP transformation and how many steps to perform, in terms of the lambda window size. MD setting, e.g., time step, cutoff- and switching-distance, and output frequencies can be updated. Finally, the number of FEP simulations to perform in the virtual screening experiment can be modified.

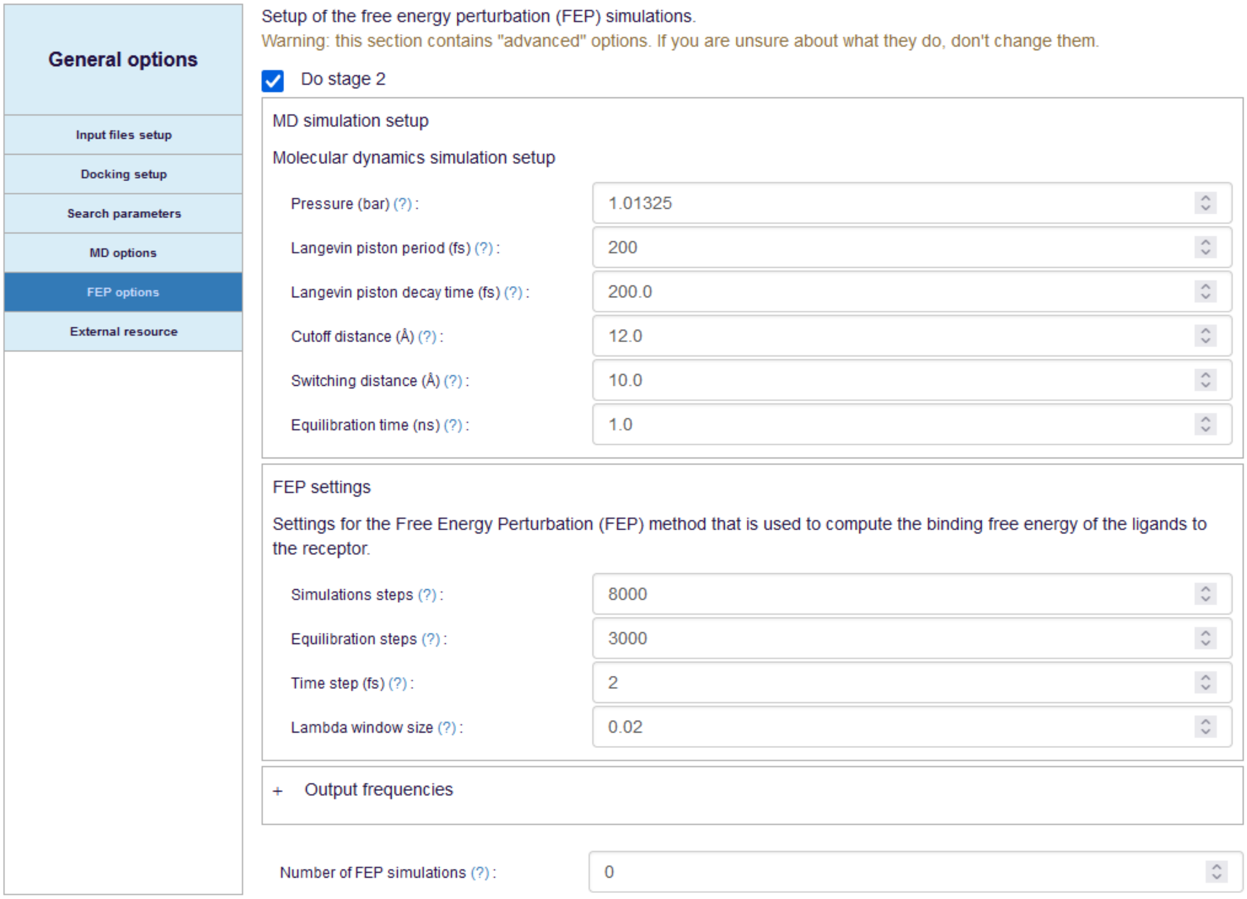


Figure S5: Sub-menu allowing to modify how the FEP simulations are performed. Typical MD settings, e.g., time step, system temperature and pressure, and output frequencies can be defined. Additionally, the quality of the FEP simulations can be adjusted by the number of equilibration and simulation steps performed in each step of the FEP transformation.

# ALISE's ranked results for the HIV protease case study

Table S1: ligand binding energies (kcal/mol) from docking stage

| Rank | Ligand ID | Binding energy |
|---|---|---|
| 1 | 28588_14254 | -11.8 |
| 2 | 28587_617 | -11.7 |
| 3 | 28588_10370 | -11.7 |
| 4 | 28588_1736 | -11.7 |
| 5 | 28587_791 | -11.6 |
| 6 | 28588_10096 | -11.6 |
| 7 | 28588_4335 | -11.6 |
| 8 | 28588_7617 | -11.6 |
| 9 | 28587_616 | -11.5 |
| 10 | 28588_10029 | -11.5 |
| 11 | 28588_13129 | -11.5 |
| 12 | 28588_1692 | -11.5 |
| 13 | 28588_13446 | -11.4 |
| 14 | 28588_984 | -11.4 |
| 15 | 28587_375 | -11.3 |
| 16 | 28588_11817 | -11.3 |
| 17 | 28588_11892 | -11.3 |
| 18 | 28588_13213 | -11.3 |
| 19 | 28588_5283 | -11.3 |
| 20 | 28588_6189 | -11.3 |
| 21 | 28588_9684 | -11.3 |
| 22 | 28588_9984 | -11.3 |
| 23 | 28587_508 | -11.2 |
| 24 | 28588_10030 | -11.2 |
| 25 | 28588_10114 | -11.2 |
| 26 | 28588_1306 | -11.2 |
| 27 | 28588_13570 | -11.2 |
| 28 | 28588_14259 | -11.2 |
| 29 | 28588_1726 | -11.2 |
| 30 | 28588_5977 | -11.2 |
| 31 | 28588_6829 | -11.2 |
| 32 | 28588_9961 | -11.2 |
| 33 | 28587_171 | -11.1 |
| 34 | 28587_510 | -11.1 |
| 35 | 28588_10084 | -11.1 |
| 36 | 28588_12812 | -11.1 |
| 37 | 28588_128 | -11.1 |
| 38 | 28588_1689 | -11.1 |
| 39 | 28588_4088 | -11.1 |
| 40 | 28588_4338 | -11.1 |
| 41 | 28588_7600 | -11.1 |
| 42 | 28588_9941 | -11.1 |
| 43 | 28587_1188 | -11.0 |
| 44 | 28587_1189 | -11.0 |
| 45 | 28587_1294 | -11.0 |
| 46 | 28587_376 | -11.0 |
| 47 | 28587_518 | -11.0 |
| 48 | 28588_10004 | -11.0 |
| 49 | 28588_10063 | -11.0 |
| 50 | 28588_10073 | -11.0 |
| 51 | 28588_11768 | -11.0 |
| 52 | 28588_12938 | -11.0 |
| 53 | 28588_13332 | -11.0 |
| 54 | 28588_13448 | -11.0 |
| 55 | 28588_13449 | -11.0 |
| 56 | 28588_215 | -11.0 |
| 57 | 28588_4395 | -11.0 |
| 58 | 28588_5599 | -11.0 |
| 59 | 28588_803 | -11.0 |
| 60 | 28588_983 | -11.0 |
| 61 | 28588_9991 | -11.0 |
| 62 | 28588_9997 | -11.0 |
| 63 | 28587_441 | -10.9 |
| 64 | 28587_507 | -10.9 |
| 65 | 28587_509 | -10.9 |
| 66 | 28587_517 | -10.9 |
| 67 | 28588_105 | -10.9 |
| 68 | 28588_11718 | -10.9 |
| 69 | 28588_11818 | -10.9 |
| 70 | 28588_12258 | -10.9 |
| 71 | 28588_12537 | -10.9 |
| 72 | 28588_1311 | -10.9 |
| 73 | 28588_13178 | -10.9 |
| 74 | 28588_13559 | -10.9 |

Table S1 (continued): ligand binding energies (kcal/mol) from docking stage

| Rank | Ligand ID | Binding energy | Rank | Ligand ID | Binding energy |
|---|---|---|---|---|---|
| 75 | 28588_14251 | -10.9 | 116 | 28588_9958 | -10.8 |
| 76 | 28588_1723 | -10.9 | 117 | 28587_115 | -10.7 |
| 77 | 28588_2127 | -10.9 | 118 | 28587_125 | -10.7 |
| 78 | 28588_2487 | -10.9 | 119 | 28587_388 | -10.7 |
| 79 | 28588_3491 | -10.9 | 120 | 28587_43 | -10.7 |
| 80 | 28588_3496 | -10.9 | 121 | 28587_440 | -10.7 |
| 81 | 28588_3887 | -10.9 | 122 | 28587_442 | -10.7 |
| 82 | 28588_3995 | -10.9 | 123 | 28587_826 | -10.7 |
| 83 | 28588_4396 | -10.9 | 124 | 28587_856 | -10.7 |
| 84 | 28588_4397 | -10.9 | 125 | 28587_859 | -10.7 |
| 85 | 28588_5689 | -10.9 | 126 | 28588_10005 | -10.7 |
| 86 | 28588_5775 | -10.9 | 127 | 28588_10110 | -10.7 |
| 87 | 28588_6828 | -10.9 | 128 | 28588_11107 | -10.7 |
| 88 | 28587_1135 | -10.8 | 129 | 28588_11113 | -10.7 |
| 89 | 28587_20 | -10.8 | 130 | 28588_11472 | -10.7 |
| 90 | 28587_443 | -10.8 | 131 | 28588_11482 | -10.7 |
| 91 | 28587_465 | -10.8 | 132 | 28588_11744 | -10.7 |
| 92 | 28587_466 | -10.8 | 133 | 28588_12729 | -10.7 |
| 93 | 28587_854 | -10.8 | 134 | 28588_13088 | -10.7 |
| 94 | 28588_10037 | -10.8 | 135 | 28588_13094 | -10.7 |
| 95 | 28588_10810 | -10.8 | 136 | 28588_13222 | -10.7 |
| 96 | 28588_11453 | -10.8 | 137 | 28588_13296 | -10.7 |
| 97 | 28588_11728 | -10.8 | 138 | 28588_13432 | -10.7 |
| 98 | 28588_11924 | -10.8 | 139 | 28588_13642 | -10.7 |
| 99 | 28588_12531 | -10.8 | 140 | 28588_13921 | -10.7 |
| 100 | 28588_1269 | -10.8 | 141 | 28588_14230 | -10.7 |
| 101 | 28588_12942 | -10.8 | 142 | 28588_14235 | -10.7 |
| 102 | 28588_13058 | -10.8 | 143 | 28588_14382 | -10.7 |
| 103 | 28588_13135 | -10.8 | 144 | 28588_1605 | -10.7 |
| 104 | 28588_13462 | -10.8 | 145 | 28588_2126 | -10.7 |
| 105 | 28588_13736 | -10.8 | 146 | 28588_3886 | -10.7 |
| 106 | 28588_14261 | -10.8 | 147 | 28588_5874 | -10.7 |
| 107 | 28588_14267 | -10.8 | 148 | 28588_5906 | -10.7 |
| 108 | 28588_14397 | -10.8 | 149 | 28588_7262 | -10.7 |
| 109 | 28588_1742 | -10.8 | 150 | 28588_7603 | -10.7 |
| 110 | 28588_4332 | -10.8 | 151 | 28588_7609 | -10.7 |
| 111 | 28588_4336 | -10.8 | 152 | 28588_7885 | -10.7 |
| 112 | 28588_4337 | -10.8 | 153 | 28588_806 | -10.7 |
| 113 | 28588_5781 | -10.8 | 154 | 28588_807 | -10.7 |
| 114 | 28588_8637 | -10.8 | 155 | 28588_9490 | -10.7 |
| 115 | 28588_9942 | -10.8 | 156 | 28587_1024 | -10.6 |

Table S1 (continued): ligand binding energies (kcal/mol) from docking stage

| Rank | Ligand ID | Binding energy |
|---|---|---|
| 157 | 28587_114 | -10.6 |
| 158 | 28587_1156 | -10.6 |
| 159 | 28587_21 | -10.6 |
| 160 | 28587_387 | -10.6 |
| 161 | 28587_46 | -10.6 |
| 162 | 28587_738 | -10.6 |
| 163 | 28587_766 | -10.6 |
| 164 | 28587_82 | -10.6 |
| 165 | 28587_836 | -10.6 |
| 166 | 28587_837 | -10.6 |
| 167 | 28588_10060 | -10.6 |
| 168 | 28588_10112 | -10.6 |
| 169 | 28588_10691 | -10.6 |
| 170 | 28588_10763 | -10.6 |
| 171 | 28588_11105 | -10.6 |
| 172 | 28588_11489 | -10.6 |
| 173 | 28588_11729 | -10.6 |
| 174 | 28588_11762 | -10.6 |
| 175 | 28588_12055 | -10.6 |
| 176 | 28588_12220 | -10.6 |
| 177 | 28588_12478 | -10.6 |
| 178 | 28588_12715 | -10.6 |
| 179 | 28588_12750 | -10.6 |
| 180 | 28588_12963 | -10.6 |
| 181 | 28588_1320 | -10.6 |
| 182 | 28588_13318 | -10.6 |
| 183 | 28588_13372 | -10.6 |
| 184 | 28588_13461 | -10.6 |
| 185 | 28588_13523 | -10.6 |
| 186 | 28588_13592 | -10.6 |
| 187 | 28588_13596 | -10.6 |
| 188 | 28588_13849 | -10.6 |
| 189 | 28588_13920 | -10.6 |
| 190 | 28588_14239 | -10.6 |
| 191 | 28588_14268 | -10.6 |
| 192 | 28588_1501 | -10.6 |
| 193 | 28588_1789 | -10.6 |
| 194 | 28588_1832 | -10.6 |
| 195 | 28588_1833 | -10.6 |
| 196 | 28588_191 | -10.6 |
| 197 | 28588_2141 | -10.6 |
| 198 | 28588_2372 | -10.6 |
| 199 | 28588_2840 | -10.6 |
| 200 | 28588_2856 | -10.6 |

Table S2: ligand binding energies (kcal/mol) from MD stage

| Rank | Ligand ID | Binding energy |
|---|---|---|
| 1 | 28588_215 | -46.99 |
| 2 | 28588_10084 | -45.99 |
| 3 | 28588_10037 | -44.34 |
| 4 | 28588_13449 | -44.25 |
| 5 | 28588_1723 | -43.56 |
| 6 | 28587_616 | -43.26 |
| 7 | 28588_9991 | -42.47 |
| 8 | 28587_509 | -41.27 |
| 9 | 28587_466 | -40.57 |
| 10 | 28587_1189 | -40.44 |
| 11 | 28587_854 | -40.37 |
| 12 | 28588_9961 | -40.37 |
| 13 | 28587_791 | -40.15 |
| 14 | 28588_3491 | -38.73 |
| 15 | 28588_14254 | -38.24 |
| 16 | 28587_1294 | -37.93 |
| 17 | 28588_9684 | -37.92 |
| 18 | 28588_1306 | -37.09 |
| 19 | 28588_7617 | -36.48 |
| 20 | 28588_10810 | -36.28 |
| 21 | 28588_4335 | -34.64 |
| 22 | 28588_9941 | -34.49 |
| 23 | 28588_14251 | -33.44 |
| 24 | 28588_5689 | -33.42 |
| 25 | 28588_11453 | -32.84 |
| 26 | 28587_1188 | -32.31 |
| 27 | 28588_6828 | -32.25 |
| 28 | 28588_13448 | -31.71 |
| 29 | 28588_11768 | -31.47 |
| 30 | 28588_11817 | -31.36 |
| 31 | 28588_1692 | -31.26 |
| 32 | 28588_9984 | -30.87 |
| 33 | 28587_507 | -30.44 |
| 34 | 28588_4088 | -29.34 |
| 35 | 28588_12537 | -27.99 |
| 36 | 28588_7600 | -27.89 |
| 37 | 28587_375 | -27.35 |
| 38 | 28588_5599 | -27.03 |
| 39 | 28588_983 | -26.95 |
| 40 | 28587_376 | -26.60 |
| 41 | 28588_105 | -25.40 |
| 42 | 28588_12531 | -25.10 |
| 43 | 28588_11924 | -23.59 |
| 44 | 28587_465 | -23.11 |
| 45 | 28588_13332 | -22.69 |
| 46 | 28588_13446 | -22.59 |
| 47 | 28588_11818 | -21.48 |
| 48 | 28588_13213 | -21.27 |
| 49 | 28587_1135 | -20.93 |
| 50 | 28588_12812 | -20.67 |

Table S3: ligand binding free energies (kcal/mol) from FEP stage

| Rank | Ligand ID | Binding energy |
|---|---|---|
| 1 | 28588_215 | -42.61 |
| 2 | 28587_466 | -40.01 |
| 3 | 28587_1189 | -31.89 |
| 4 | 28588_14254 | -31.04 |
| 5 | 28588_9991 | -25.54 |
| 6 | 28587_616 | -24.40 |
| 7 | 28587_791 | -23.33 |
| 8 | 28588_9961 | -21.88 |
| 9 | 28587_854 | -21.46 |
| 10 | 28588_1723 | -20.41 |
| 11 | 28588_3491 | -19.71 |
| 12 | 28588_10037 | -18.02 |
| 13 | 28588_13449 | -15.70 |
| 14 | 28588_10084 | -14.73 |
| 15 | 28587_509 | -13.37 |

# References


(1) Kim, S.; Thiessen, P. A.; Bolton, E. E.; Chen, J.; Fu, G.; Gindulyte, A.; Han, L.; He, J.; He, S.; Shoemaker, B. A.; Wang, J.; Yu, B.; Zhang, J.; Bryant, S. H. PubChem substance and compound databases. *Nucleic Acids Res.* **2016**, *44*, D1202–D1213.

(2) Kim, S.; Chen, J.; Cheng, T.; Gindulyte, A.; He, J.; He, S.; Li, Q.; Shoemaker, B. A.; Thiessen, P. A.; Yu, B.; Zaslavsky, L.; Zhang, J.; Bolton, E. E. PubChem in 2021: new data content and improved web interfaces. *Nucleic Acids Res.* **2021**, *49*, D1388–D1395.

(3) Kim, S.; Thiessen, P. A.; Bolton, E. E.; Bryant, S. H. PUG-SOAP and PUG-REST: web services for programmatic access to chemical information in PubChem. *Nucleic Acids Res.* **2015**, *43*, W605–W611.

(4) Kim, S.; Thiessen, P. A.; Cheng, T.; Yu, B.; Bolton, E. E. An update on PUG-REST: RESTful interface for programmatic access to PubChem. *Nucleic Acids Res.* **2018**, *46*, W563–W570.

(5) Belford, R. *How to Search PubChem for Chemical Information (Part 2)*; 2019; accessed: 2021-04-30.

(6) Bajusz, D.; Rácz, A.; Héberger, K. Why is Tanimoto index an appropriate choice for fingerprint-based similarity calculations? *J. Cheminf.* **2015**, *7*, 1–13.

(7) Weininger, D. SMILES, a chemical language and information system. 1. Introduction to methodology and encoding rules. *J. Chem. Inf. Comput. Sci.* **1988**, *28*, 31–36.

(8) Harris, R.; Olson, A. J.; Goodsell, D. S. Automated prediction of ligand-binding sites in proteins. *Proteins: Struct., Funct., Bioinf.* **2008**, *70*, 1506–1517.

(9) Morris, G. M.; Goodsell, D. S.; Halliday, R. S.; Huey, R.; Hart, W. E.; Belew, R. K.; Olson, A. J. Automated docking using a Lamarckian genetic algorithm and an empirical binding free energy function. *J. Comput. Chem.* **1998**, *19*, 1639–1662.

(10) Morris, G. M.; Huey, R.; Lindstrom, W.; Sanner, M. F.; Belew, R. K.; Goodsell, D. S.; Olson, A. J. AutoDock4 and AutoDockTools4: Automated docking with selective receptor flexibility. *J. Comput. Chem.* **2009**, *30*, 2785–2791.
